# Improvement Research Priorities: USA Survey and Expert Consensus

**DOI:** 10.1155/2013/695729

**Published:** 2013-08-18

**Authors:** Kathleen R. Stevens, John Ovretveit

**Affiliations:** ^1^Academic Center for Evidence-Based Practice, School of Nursing, University of Texas Health Science Center San Antonio, San Antonio, TX 78229, USA; ^2^Steering Council Member, Improvement Science Research Network, USA; ^3^Medical Management Centre, The Karolinska Institutet, 17177 Stockholm, Sweden

## Abstract

The purpose of this study was to identify stakeholder views about national priorities for improvement science and build agreement for action in a national improvement and implementation research network in the USA. This was accomplished using three stages of identification and consensus. (1) Topics were identified through a multipronged environmental scan of the literature and initiatives. (2) Based on this scan, a survey was developed, and stakeholders (*n* = 2,777) were invited to rate the resulting 33-topic, 9-category list, via an online survey. Data from 560 respondents (20% response) were analyzed. (3) An expert panel used survey results to further refine the research priorities through a Rand Delphi process. Priorities identified were within four categories: care coordination and transitions, high-performing clinical systems and microsystems improvement approaches, implementation of evidence-based improvements and best practices, and culture of quality and safety. The priorities identified were adopted by the improvement science research network as the research agenda to guide strategy. The process and conclusions may be of value to quality improvement research funding agencies, governments, and research units seeking to concentrate their resources on improvement topics where research is capable of yielding timely and actionable answers as well as contributing to the knowledge base for improvement.

## 1. Introduction

Improvement science is an emerging multidiscipline which overlaps with other fields such as implementation science and conventional medical research as noted by Wensing and others [1]. Experts point out that the evidence base for the science spans controlled trials of interventions with patients, providers, and organizations, to small scale rapid cycle testing of improvement changes by local project teams [2]. A healthy debate in the literature focuses on the type and strength of evidence which might form the scientific basis and whether the aim should be to build a knowledge domain with characteristics distinct from other sciences [3–5]. 

Largely separate to these debates, healthcare organizations and governments globally are implementing different ideas and interventions which hold promise for improving quality, safety, and performance [6]. Research of different types can contribute to more effective choice and implementation of improvements, but resources are limited, and expertise in this field is scarce. There is a case for concentrating research resources on investigating targeted problems and potential solutions. If researchers and funders were to concentrate efforts and resources, then which topics and improvement strategies should be the focus and which criteria should be used to set priorities? Should choices be made on the likelihood of the question being answerable by current methods? How much weighting should be given to the likely practical value of the findings for action and how much should be given to developing methods, measures, and theories? 

This project aimed to identify national priorities for improvement science and create an agenda to focus and guide researchers and funders. One motive for this work was a felt need by a USA national improvement research network to create a strategy for improvement research and a consensus about priorities. Founders of this network emphasized collaboration between researchers in different centers and services so as to maximize the use and value of improvement research [7, 8]. The leaders took the view that consensus-based research priorities would serve as a common rallying point for improvement scientists and clinical leaders to collaborate around common research goals. 

## 2. Materials and Methods

The three-stage approach used to identify improvement topics and develop consensus about priorities is outlined in Table 1 and included (1) environmental scan to identify improvement topics, (2) development, refinement, and administration of a stakeholder survey, and (3) consensus development by an expert panel. Further details of methods are available in the resource document available from the authors, which also provides guidance to others seeking to use a similar process. 

### 2.1. Environmental Scan to Identify Topics

A review identified improvement topics from healthcare literature. In addition, topics were identified from guidance and requirements issued in regulatory and accreditation criteria (e.g., The Joint Commission goals) [9]. Additional improvement topics were identified from challenges identified in improvement innovations (e.g., AHRQ Health Care Innovations Exchange) [10], national improvement campaigns (e.g., IHI 100 K Lives Campaign) [11], and discussions with improvement leaders. 

### 2.2. Survey Development, Refinement, and Administration

A stakeholder survey about improvement research priorities was developed through several steps. First, a list of 33 topics identified from the environmental scan were used in an initial paper-based survey and administered to healthcare professionals attending a quality improvement conference event (*n* = 320, response rate = 62%). Descriptive statistics of these responses showed that all of the 33 topics were rated as “highly important” (on a 0–10 point scale). Respondents also suggested additional topics, all of which were logically grouped within the existing categories. The revised survey organized the 33 topics into 9 categories with a 1–6 point rating scale.

*Highlights of Survey to Set Research Priorities for Improvement Science*

*Categories and Improvement Strategies*

1. Process improvement in clinical care
  - Evidence-based practice in clinical care
  - Integration of best practices into clinical routines
  - Checklists and other care improvement tools
  - Process improvement techniques and tools (e.g., plan-do-study-act, root cause analysis, and Six Sigma)
2. Systems and microsystems
  - Workplace environment and quality improvement
  - Climates for change and learning organizations
  - Innovation for improvement
  - Adoption of best practices (hardwiring change)
  - High-reliability organization concepts in acute care settings
3. Patient safety
  - Culture of patient safety (e.g., shared willingness to report and learn from errors, open communication, leadership support)
  - Prevention of targeted patient safety incidents (e.g., falls, medication errors)
4. Patient-centered care
  - Patient and family activation and engagement
  - Patient-centered care and patient advocacy
5. Care coordination
  - Handoffs and transitions *within* the hospital
  - Handoffs and transitions *across* healthcare settings
6. Quality indicators (e.g., performance and outcome measures)
  - Quality indicator sets (e.g., *National Database of Nursing Quality Indicators*)
  - Reliable metrics for measuring improvement
  - Reports to the public on quality and safety (transparency)
  - Feedback and dashboards to guide performance
  - Baseline and follow-up measures to assess impact of improvement
  - Measurement of total system processes
7. Policy, regulation, and recognition programs
  - Impact of healthcare policy issues (e.g., public reporting, pay for performance)
  - Economic impact of improvement processes
  - Programs of excellence impact on patient outcomes (e.g., Magnet Recognition)
  - Economic impact of healthcare regulations on costs and outcomes
8. Workforce preparation and competencies
  - New competencies for quality improvement and patient safety
  - Redesign of clinical roles (e.g., clinical nurse leaders)
  - Appropriate staffing levels
  - Frontline provider engagement in quality and safety (e.g., Transforming Care at the Bedside)
  - Team performance and interprofessional communication (e.g., standardized team training)
  - Disruptive behavior management
9. Technology
  - Technology applications in clinical care (e.g., electronic decision support tools, smart pumps)
  - Integration of technology applications into clinical care.

Over the next four months, 12 quality experts from an improvement research network advisory board contributed to content review and refining and clarifying wording. An online survey was piloted with five additional experts in healthcare improvement. The respondents were subsequently debriefed via telephone to assess clarity of the online survey and offer changes. This led to a revision of the 1–6-point scale to the 1–4-scale used in the subsequent survey of healthcare professionals as stakeholders. The final survey is available as online Supplementary Material at http://dx.doi.org/10.1155/2013/695729. 

The aim was to survey a range of stakeholders with interest in quality improvement research, including researchers, academics, clinicians, administrators, health service personnel, and others. Because no single sampling frame exists for this group, to achieve representation from multiple disciplines and settings, the target sample for the survey was compiled from several lists. These lists included purchased lists from professional societies (e.g., healthcare executive associations, health scientist groups), a commercial list of multiple disciplines focused on improvement, and an internal improvement practice-and-research list which had been built over 10 years from a variety of sources. While those included in the sampling frame were selected through a variety of approaches, because of their affiliations, they were deemed to share a common interest in evidence-based quality improvement, delivery improvement, and patient safety. Characteristics of the final sampling frame were reviewed to assure that it included directors and health professionals associated with scientific groups, clinical leaders, evidence-based practice leaders, and clinical directors, managers, and administrators; the list also included persons associated with excellence recognition programs (e.g., Malcolm Baldrige Quality Award and Magnet hospital recognition), individuals involved in the Agency for Healthcare Research and Quality (AHRQ) Health Care Innovations Exchange (a database of tested healthcare innovations implemented in the US and Canada) [10], leaders on healthcare research society boards, editors of quality and patient safety journals, academic deans and faculty, and others attending conferences on interprofessional evidence-based quality improvement.

The online stakeholder survey was administered following the Dillman method [12]. This involved a prenotification email three days before the survey; survey invitation email and survey link; then, email reminders at 7 days and 14 days after survey initiation.

Methods used to analyze results included analysis of respondent characteristics (Table 2, in the “results” section below); “home” institutions; and ratings of topic importance. Topics rated as “very important” formed the basis of the next phase of consensus development.

### 2.3. Consensus Development of an Improvement Research Agenda

A Delphi method, as advanced by Rand, was used to develop consensus about research priorities [13], using an expert panel. The panel was made up of 14 experts representing clinical, academic, science, and management roles in the USA and one international expert. Two months prior to an in-person meeting, the panel completed the stakeholder survey described previously. Results of the panel's survey and of the stakeholder survey were discussed and processed by the group during the one-day, professionally facilitated meeting. The meeting involved five steps.

**Step 1**

Panel members were presented: (1) top ten priorities identified by the stakeholder survey and (2) premeeting priorities selected by the panel on the same survey.

**Step 2**

Panel members discussed their opinions on the importance of various improvement topics. Members developed a list of criteria for assessing the importance of research topics, described in the Results section.

**Step 3**

Members casted a second vote by completing a slightly modified version of the stakeholder survey and ranked their “top three” improvement strategies. Project analysts compiled these results, identifying the topics the panel had identified as most important.

**Step 4**

The facilitator reported results from the second vote and facilitated further discussion. The highest priority topics were identified using two criteria: number of panel members ranking the item as “top three” and number of panel members rating the item as “very important.”

**Step 5**

The panel debated not only the top research priorities but also the best way to categorize them. Panel members reviewed the top priorities identified in Step 4 and derived a list of four research priorities.

## 3. Results and Discussion

### 3.1. Analysis of Respondents

Email invitations to complete the internet survey were sent to 2,777 stakeholders; 560 completed the survey (20% response rate). Demographic characteristics (Table 2) reflected that respondents were 46% researchers, 34% clinical administrators or managers, and 13% frontline clinicians. Eighty-four percent had over 11 years of experience and 96% held an advanced degree. Forty-five percent were university-based professors. Of those responding, 85% were nurses and 16% held medical doctorates. Based on desired target sample characteristics, respondents represented the intended target sample. The preponderance held advanced degrees in experienced careers and were in leadership positions. For the scientific sector, key stakeholder researchers (46%) and university professors (45%) were well represented. Clinical administrators and managers (total 47%) were also well represented. The multidisciplinary makeup of the respondents (RNs = 84%; MDs = 16%) roughly aligned with national proportions (RNs = 79% [14]; MDs = 21% [15]).

### 3.2. Analysis of Responses

Survey results showed that quality improvement initiatives were strongly supported at respondents' home institutions. Of the respondents, 92% percent agreed that their institutions' healthcare professionals are expected to improve processes and systems of care; 88% noted that the actions of leaders show that patient safety and quality improvement are top priorities; 84% agreed that clinicians engage in quality improvement and patient safety strategies in their daily work; and 77% agreed that clinical staff feels free to suggest changes and new programs. 

#### 3.2.1. Highest Rated Topics and Variations in Topic Ratings

Respondents rated 87% of the 33 topics as “very important” or “important.” Table 3 displays the summary of use of each scale descriptor. Few topics were rated “somewhat important” (12%) or “not important” (1%).

Table 4 presents the ten topics most frequently rated as “very important.” Topics least frequently rated as “very important” fell into two categories: (1) policy, regulation, and recognition programs and (2) workforce preparation and competencies.

There were variations in respondent ratings of the 33 topics in the 9 categories. For example, a large majority of respondents rated two of the four topics in the category “process improvement in clinical care” as highly important: “evidence-based practice in clinical care” (68%) and “integration of best practices into clinical routines” (73%). By contrast, fewer respondents rated the other two topics in this category as highly important: “checklists and other tools” (44%) and “process improvement techniques and tools” (35%). Additionally, in the “patient-centered care” category, about half of the respondents rated both of the two topics as “very important.” In the “patient safety” category, more than two-thirds considered the two topics to be “very important.” This contrasted with ratings of the four topics in the “policy, regulation, and recognition programs” category; these ratings ranged from 39% to 49%.

#### 3.2.2. Variations in Ratings by Respondent Characteristics

Two respondent subgroupings, “researcher/scientists” (46%) and “nonresearchers” (54%), showed similarity in selection of the top ten improvement topics. Eight topics were in the top ten for both groups. Career experience subgroups (5 or fewer years; 6–15 years; 16–20 years; more than 20 years) showed similarity in only five topics ranked in the top ten. 

The analysis also showed differences in the top ten topics rated across education level. Five topics were in the top ten for all groups. Notably, respondents with more experience and higher education were more likely to rate as “very important” items in the workforce preparation and competencies categories.

Forty-three percent of respondents suggested a total of 515 additional topics not listed in the original survey. These additional topics focused on specific populations (such as elderly), different care settings, and specific design strategies. All were conceptually grouped into the nine categories in the survey.

#### 3.2.3. Consensus Prioritization Results

Criteria formulated in Step 3 for deciding priority topics were as follows: potential impact on patient health and safety; quick payoff; cost effectiveness; presence of data gap (i.e., critical need for evidence-based information); practice community's priorities and concerns; fundability; simplicity; likelihood of success/failure; diversity of focus; greatest areas of uncertainty; current issues within practice environments; and likelihood of clinician engagement. 

In this phase, experts used two criteria to identify the highest priority improvement topics: (1) the number of panel members ranking the item in the “top three” and (2) the number of panel members rating the item as “very important.” Both approaches pointed to the same top two priorities: “integration of best practices into clinical routines” (survey item number 2) and “frontline provider engagement in quality and safety” (survey item number 29). Other items rated as “very important” by the majority of panel included “handoffs and transitions within the hospital and across healthcare settings;” “workplace environment and quality improvement;” “climates for change and learning organizations;” and “prevention of targeted patient safety incidents.”

During Step 5, each member reviewed the top priority topics identified in Step 4 and derived a list of three or four research priorities. During this process, the panel noted the difficulty of comparing such diverse topics as coordination of transitions in care, effectiveness, and efficiency of various methods and models for best practices, evidence-based practices for outcome improvement, and improved organizational environments. They noted that quality and safety require efforts on multiple fronts. Discussion then focused on separating the overlapping areas and differentiating distinct subject categories from the prioritized subjects. This resulted in members recommending four priority research topics: care coordination and transitions, approaches to improvement used by high-performing clinical systems and microsystems, evidence-based quality improvement and best practices, and culture of quality and safety. To provide further detail and add meaning to each category, priority topics and examples of strategies and research issue were developed for each category.  Table 5 presents the resulting improvement research agenda.

## 4. Discussion

This study produced the first national stakeholder-informed research agenda for the study of improvement and implementation strategies. The consensus priorities highlight the most important and urgent needs in improving knowledge as identified by clinical and academic scholars, leaders, and change agents in acute healthcare settings. The expert panel approach was successful in building on stakeholder survey results to further define and prioritize a research agenda that reflected consensus. Final priorities were crafted into a statement which the experts considered would be understood by those they represented and thus could be effectively communicated to the larger group of stakeholders. 

This research agenda reflects knowledge needs in general areas of improvement; a more specific research agenda would provide clear guidance for scientists and clinicians in the field. Challenges in creating such an agenda arise from several sources. First, the fields of improvement and implementation science are new and, as such, lack common terminology. At the same time, other related fields such as translational science and knowledge translation can also share many scientific priorities and overlaps in the goal of quality healthcare. The improvement research agenda provides a starting point for building interchange with knowledge domains, common frameworks, and scientific capacity across these fields. This method could be used by other groups both to identify priorities and develop commitment to a research agenda in new fields. The consensus topics will be of interest to those working in overlapping and related fields, including “translational science,” “implementation research,” “healthcare innovation,” and “service delivery research.”

Any survey and consensus process reflect characteristics of the participants and the methods used. This study resulted in national, interprofessional consensus across those who took part in the various stages of the process of providing and interpreting information. Because improvement stakeholders are not a homogeneous group and are from many disciplines and traditions, the sampling frame was created from multiple sources. It likely included some that did not represent the target population; this may have affected our response rate. The large sampling frame did result in a sizeable number of respondents from academic and clinical settings (almost equally distributed), multiple professions, and a range of experience and education. Because only a few demographic variables were collected from the respondents, it is not possible to provide a detailed profile of the respondents. 

A source of bias in this study arises from the early and evolving state of improvement science. Because concepts of improvement that were used in this work are not yet well defined in this emerging field, usage and meaning of terms are not precise. Terms such as “patient centered care” and “microsystems” may be defined in different ways. This lack of common terminology presented obstacles to accurate communication in the surveys used and, to a lesser extent, in the in-person interactions across multiple disciplines.

This study produced research priorities reflecting not only the rapidly emerging field of improvement science but also the perspectives of stakeholders who are new partners in improving care and patient safety. Perspectives of university-based researchers and clinical practitioners and managers regarding research priorities are affected by the incentives and core activities in each setting. This study did not detect wide disparities between these two groups; rather, it provided some indications that the perspectives of research/scientists and nonresearch/scientists were similar in their top ten priority topics. This could be due to the approach used to identify the study sample. Efforts to identify quality improvement stakeholders, whether researchers or clinicians, resulted in inviting respondents from both groups that already shared a common focus. In addition, as the consensus moved to the expert panel, the improvement science focus was further sharpened.

The picture reported above is representative of perceptions of the informed persons and experts on the importance of quality improvement research in the USA at that point in time. With rapid changes occurring in USA healthcare delivery, perceptions of the respondents may have changed since the survey. Following the network's adoption of the priority statements, they have been continuously monitored and annually reviewed by the international steering group of the improvement research network, assuring continued alignment with contemporary needs.

What can improvement leaders and researchers in other countries learn from this process and the findings? First, that there are many different groups with an interest in improvement science and related fields. These priorities provide a sound reference point for initial discussions across improvement, implementation, health delivery, and translational sciences. Second, that identifying and communicating with all who have an interest in and can contribute to improvement science and research may be more difficult than expected because of the lack of clear constructs and classifications. This can make building consensus about priorities difficult to achieve, but doing so also helps build scientific communities and networks. Thirdly, differences in views about appropriate research methods and approaches may emerge from fields that traditionally use randomized control trials and differently used terms across groups.

This research agenda can prompt a reframing of the current quality improvement research paradigm to include collaborative, rigorous studies of strategies across academic-practice partnerships. Articulation of top priorities can help to develop common terminology with which to advance discussion between academic and clinical partners about the kinds of studies needed to improve care and patient safety.

This initial formulation of research priorities highlights several remaining challenges. The first is to design rigorous scientific investigations of specific quality improvement initiatives that can be adopted in healthcare. The expert panel considered how to articulate research priorities that would be broad enough to encompass critical areas of research, yet sufficiently specific to guide the identification of actual research topics. Ultimately, the panel defined four general areas for research, provided descriptions, and suggested examples but stopped short of detailing actionable research questions or hypotheses. Toward this end, four multisite network demonstration projects are currently underway as described by the research network [16].

A second challenge is availability of scientists who are prepared to test quality improvement interventions. As education bodies address this challenge (e.g., American Association of Colleges of Nursing and American Association of Medical Colleges), there is also the need to innovate in research methods and designs to be more responsive to practical and scientific criticisms of some improvement research.

A final challenge is the lack of a universally accepted vocabulary to ensure clear communication about improvement concepts. While the improvement research agenda is general, it does provide a basis for classifying improvement strategies to be tested. 

The process described and the resulting priority statements have led to decisions about resource investment as research projects were selected, developed, and conducted in the last 18 months addressing Priorities B-Microsystems and Priority D-Learning Organizations and Culture of Safety. The next 18 months will further address Priority B and D as well as additional research projects addressing Priority A-Care Transitions, and Priority C-Evidence-Based Best Practice is discussed further on the research network website (http://www.isrn.net/) [16].

## 5. Conclusions

The priorities identified were adopted by the improvement science research network to guide their strategy. The process and conclusions may be of value to quality improvement research funding agencies, governmental units, and research units seeking to concentrate their resources on topics where research is capable of yielding timely, actionable answers.

## Supplementary Material

This survey was used to gather stakeholder input to establish consensus on research priorities in improvement science. The survey contains 33 improvement topics organized into 9 categories using a Likert scale of 1 to 4 on the construct of “importance.” It was designed from stakeholder input and systematically refined to be used online. Items and categories were initially identified from an environmental scan to identify improvement topics and categories.

## Figures and Tables

**Table 1:** Three-stage process for establishing consensus on research priorities.

Stage 1	Stage 2	Stage 3
Topic identification	Stakeholder survey	Priority consensus
Topics identified through broad environmental scans of healthcare literature, regulatory and accreditation criteria, innovation challenges, national campaigns, and discussions with leaders	Survey instrument was developed and refined as follows: (i) paper-based survey data collection (*n* = 320; 62% response rate); (ii) content and clarity review (*n* = 12); (iii) online survey pilot and telephone debrief (*n* = 5) Online survey data were collected from stakeholders (*n* = 2,777; 20% response rate)	Consensus formed by Expert Panel (Delphi process) (*n* = 14) (i) Expert panel completed online survey prior to meeting (ii) Criteria for prioritization established at in-person meeting (iii) Discussions and multiple iterations occurred during in-person meeting Consensus declared by professional facilitator and group

*Result* Topics were incorporated into survey instrument	*Result* Stakeholder survey data provided foundation for consensus formation	*Result* Priority research agenda finalized and disseminated

**Table 2:** Stakeholder survey respondent characteristics (*n* = 560).

Characteristic Position(s) held*	Number (%)	Percent
Researcher/scientist	254	46
Academic faculty member	227	41
Administrator	132	24
Clinical educator	102	18
Consultant	71	13
Frontline clinician	71	13
Midlevel manager	31	6
Supervisor/coordinator	24	4
Unit manager	7	1
Other	72	13
Total (missing)* *Respondents were allowed to select all applicable	NA	NA
Years of career experience as a health professional		
More than 20 years	350	63
16–20 years	61	11
11–15 years	64	12
6–10 years	34	6
1–5 years	39	7
Less than 1 year	5	1
Total (missing)	553 (7)	100 (1)
Highest level of education		
Doctorate degree	235	47
Master's degree	151	30
Medical doctorate degree	78	16
Bachelor's degree	23	5
Other	11	2
Total (missing)	498 (62)	100 (11)

**Table 3:** Frequency (%) with which survey scale descriptors were used to rate 33 improvement topics (by 560 respondents).

Survey scale descriptor	Mean %	Median %	Min–max % (range)
Very important	51	48	28–74 (46)
Important	36	36	21–46 (25)
Somewhat important	12	12	2–27 (25)
Not important	1	1	0–5 (5)
COMBINED (important and very important)	87	87	68–98 (30)

**Table 4:** Top ten improvement topics ranked by frequency of “very important” rating (*n* = 560).

Improvement topic (ranked highest to lowest)	*N* (%) rating “very important”
Handoffs and transitions across healthcare settings	414 (74)
Integration of best practices into clinical routines	408 (73)
Culture of patient safety	386 (69)
Evidence-based practice in clinical care	381 (68)
Prevention of targeted patient safety incidents	381 (68)
Reliable metrics for measuring improvement	364 (65)
Adoption of best practices	336 (60)
Integration of technology applications into clinical care	325 (58)
Baseline and follow-up measures to assess impact of improvement	325 (58)
Handoffs and transitions within the hospital	319 (57)

**Table 5:** National improvement research agenda.

Category	Priority topics	Examples of strategies and research issues
(A) Coordination and transitions of care This category emphasizes strategies for care improvement to care processes in specific clinical conditions. At this time, care coordination and transitions of care are the key clinical focus	(i) Evaluate strategies and methods to assure coordination and continuity of care across transitions in given clinical populations (ii) Test and refine methods of handoffs and other strategies to assure safe, effective, and efficient transitions in given clinical populations	Interprofessional team performance, medication reconciliation, discharge for prevention of early readmission, patient-centered care, and measurement of targeted outcomes

(B) High-performing clinical systems and microsystems approaches to improvement This category emphasizes structure and process in clinical care and healthcare as complex adaptive systems	(i) Determine effectiveness and efficiency of various methods and models for integrating and sustaining best practices in improving care processes and patient outcomes (ii) Investigate strategies to engage frontline providers in improving quality and patient safety (iii) Evaluate strategies for preventing targeted patient safety incidents (iv) Establish reliable quality indicators to measure impact of improvement and isolate nursing care impact on outcomes	Frontline provider engagement, unit-based quality teams, factors related to uptake, adoption, and implementation, sustaining improvements and improvement processes, academic-practice partnership, and informatics solutions

(C) Evidence-based quality improvement and best practice This category emphasizes closing the gap between knowledge and practice through transforming knowledge and designating and implementing best practices	(i) Evaluate strategies and impact of employing evidence-based practice in clinical care for process and outcomes improvement (ii) Determine gaps and bridge gaps between knowledge and practice (iii) Transform evidence for practice through conducting systematic reviews, developing practice guidelines, and integrating practice into clinical decision making (iv) Develop new research methods in evidence-based quality improvement, including comparative effectiveness research and practice-based evidence	Develop and critically appraise clinical practice guidelines, adoption and spread of best practices, customization of best practices, institutional elements in adoption, defining best practice in absence of evidence, consumers in evidence-based practice, and technology-based integration

(D) Learning organizations and culture of quality and safety This category emphasizes human factors and other aspects of a system related to organizational culture and commitment to quality and safety	(i) Investigate strategies for creating organizational environments, processes that support cultures fully linked to maintaining quality, and patient safety in order to maximize patient outcomes (ii) Determine effective approaches to developing organizational climates for change, innovation, and organizational learning	Professional practice environments, protecting strategy from culture, shared decision making and governance, patient-centered models, leadership to instill values and beliefs for culture of patient safety, and organizational design (e.g., omit first-order failures)
