# Supplementary material for: Improvement Research Priorities: USA Survey and Expert Consensus
Source: Nurs Res Pract. 2013 Aug 18;2013:695729. doi: 10.1155/2013/695729 (PMC3759273; doi:10.1155/2013/695729)
Supplement: Supplementary file 1 — This survey was used to gather stakeholder input to establish consensus on research priorities in improvement science. The survey contains 33 improvement topics organized into 9 categories using a Likert scale of 1 to 4 on the construct of “importance.” It was designed from stakeholder input and systematically refined to be used online. Items and categories were initially identified from an environmental scan to identify improvement topics and categories. [file 695729.f1.pdf]

[Electronic Appendix]

Survey to Set Research Priorities for Improvement Science

INTRODUCTION TO SURVEY

Survey to Set Research Priorities for Improvement Science Copyright Stevens, 2011 Reprinted with permission.

This survey was used to gather stakeholder input to establish consensus on research priorities in improvement science. The survey contains 33 improvement topics organized into 9 categories using a Likert scale of 1 to 4 on the construct of "importance." It was designed from stakeholder input and systematically refined to be used online. Items and categories were initially identified from an environmental scan to identify improvement topics and categories.

Improvement science holds great promise for improving patient care, outcomes, and system efficiencies and thus it is important to increase the quantity and quality of improvement research. We are seeking your recommendations as a researcher, academician, clinician, or administrator on priorities for improvement research.

1. In your opinion, how important is it to study each of the following improvement strategies?

Improvement Strategies	Not important at all	Somewhat important	Important	Very important
Process Improvement in Clinical Care				
Evidence-based practice in clinical care	☐1	☐2	☐3	☐4
Integration of best practices into clinical routines	☐1	☐2	☐3	☐4
Checklists and other care improvement tools	☐1	☐2	☐3	☐4
Process improvement techniques and tools (e.g., Plan-Do-Study-Act, root cause analysis, Six Sigma)	☐1	☐2	☐3	☐4
Systems and Microsystems				
Workplace environment and quality improvement	☐1	☐2	☐3	☐4
Climates for change and learning organizations	☐1	☐2	☐3	☐4

Innovation for improvement	☐1	☐2	☐3	☐4
Adoption of best practices (hardwiring change)	☐1	☐2	☐3	☐4

High-reliability organization concepts in acute care settings	☐1	☐2	☐3	☐4
Patient Safety				
Culture of patient safety (e.g., shared willingness to report and learn from errors, open communication, leadership support)	☐1	☐2	☐3	☐4
Prevention of targeted patient safety incidents (e.g., falls, medication errors)	☐1	☐2	☐3	☐4
Patient Centered Care				
Patient and family activation and engagement	☐1	☐2	☐3	☐4
Patient-centered care and patient advocacy	☐1	☐2	☐3	☐4
Care Coordination				
Handoffs and transitions within the hospital	☐1	☐2	☐3	☐4
Handoffs and transitions across healthcare settings	☐1	☐2	☐3	☐4
Quality Indicators (e.g., performance and outcome measures)				
Quality indicator sets (e.g., *National Database of Nursing Quality Indicators)*	☐1	☐2	☐3	☐4
Reliable metrics for measuring improvement	☐1	☐2	☐3	☐4
Reports to the public on quality and safety (transparency)	☐1	☐2	☐3	☐4
Feedback and dashboards to guide performance	☐1	☐2	☐3	☐4
Baseline and follow up measures to assess impact of improvement	☐1	☐2	☐3	☐4
Measurement of total system processes	☐1	☐2	☐3	☐4
Policy, Regulation, and Recognition Programs				
Impact of healthcare policy issues (e.g., public reporting, pay for performance)	☐1	☐2	☐3	☐4
Economic impact of improvement processes	☐1	☐2	☐3	☐4

Programs of excellence impact on patient outcomes (e.g., Magnet Recognition)	☐1	☐2	☐3	☐4
Economic impact of healthcare regulations on costs and outcomes	☐1	☐2	☐3	☐4
Workforce Preparation and Competencies				
New competencies for quality improvement and patient safety	☐1	☐2	☐3	☐4
Redesign of clinical roles (e.g., clinical nurse leaders)	☐1	☐2	☐3	☐4
Appropriate staffing levels	☐1	☐2	☐3	☐4
Frontline provider engagement in quality and safety (e.g., Transforming Care at the Bedside)	☐1	☐2	☐3	☐4
Team performance and interprofessional communication (e.g., standardized team training)	☐1	☐2	☐3	☐4
Disruptive behavior management	☐1	☐2	☐3	☐4
Technology				
Technology applications in clinical care (e.g., electronic decision support tools, smart pumps)	☐1	☐2	☐3	☐4
Integration of technology applications into clinical care	☐1	☐2	☐3	☐4

2. List other improvement strategies that are important to study.

   a.

   b.

   c.

3. A national collaboration for improvement research is developing. In your opinion, what are the most important resources and supports that it should provide?

About Your Organization

Indicate typical characteristics of your work environment or affiliation by indicating your agreement with the following statements:

In my Work Environment/Affiliation…	Strongly Disagree	Disagree	Agree	Strongly Agree
The actions of leaders show that patient safety and quality improvement are top priorities	☐1	☐2	☐3	☐4
Health care professionals are expected to improve processes and systems of care	☐1	☐2	☐3	☐4
Clinicians engage in quality improvement and patient safety strategies in their daily work	☐1	☐2	☐3	☐4
Clinical staff feel free to suggest changes and new programs	☐1	☐2	☐3	☐4

About You

1. Career Experience as Health Professional	2. Type of position (*Mark all that apply*)
☐ Less than 1 year	☐ Frontline Clinician
☐ 1-5	☐ Educator-Clinical

☐ 6-10<br>☐ 11-15<br>☐ 16-20<br>☐ More than 20	☐ Faculty-Academic<br>☐ Researcher/Scientist<br>☐ Administrator<br>☐ Mid-level Manager<br>☐ Supervisor/Coordinator<br>☐ Unit Manager<br>☐ Consultant<br>☐ Other ___________________
3. Does your hospital have Magnet Recognition Status?<br>☐ Yes<br>☐ No<br>☐ Application in progress<br>☐ Not Applicable	4. Highest level of Education<br>☐ Bachelors degree<br>☐ Masters degree<br>☐ Doctorate degree<br>☐ Medical doctorate<br>☐ Other _________________

Thank you for your valuable input.
